# SOLOMON: a method for splitting a sample into equivalent subsamples in factor analysis

**DOI:** 10.3758/s13428-021-01750-y

**Published:** 2021-12-16

**Authors:** Urbano Lorenzo-Seva

**Affiliations:** Universitat Rovira i Virgili, ctra de Valls s/n, 43007 Tarragona, Spain

**Keywords:** sample splitting, replication, exploratory factor analysis, confirmatory factor analysis, Duplex method, KMO index, SPSS, R

## Abstract

Nowadays, exploratory and confirmatory factor analyses are two important consecutive steps in an overall analysis process. The overall analysis should start with an exploratory factor analysis that explores the data and establishes a hypothesis for the factor model in the population. Then, the analysis process should be continued with a confirmatory factor analysis to assess whether the hypothesis proposed in the exploratory step is plausible in the population. To carry out the analysis, researchers usually collect a single sample, and then split it into two halves. As no specific splitting methods have been proposed to date in the context of factor analysis, researchers use a random split approach. In this paper we propose a method to split samples into equivalent subsamples similar to one that has already been proposed in the context of multivariate regression analysis. The method was tested in simulation studies and in real datasets.

Factor analysis is a widely used multivariate technique that was initially proposed to explore data, especially in the context of the development and assessment of psychological tests. During the 1980s and 1990s (see the detailed explanation by Michael Browne, 2001), structural equation modeling techniques suggested that exploratory factor analysis (EFA) was a poor substitute of what was then considered to be the most highly technical and correct approach: confirmatory factor analysis (CFA). It was not until the first decade of the millennium that EFA recovered its lost prestige. Nowadays, both EFA and CFA are considered two important consecutive steps in an overall process of analysis.

The popularity of factor analysis as an analysis technic has regularly been reported (see for example, Baglin, 2014; Costello & Osborne, 2005; Fabrigar et al., 1999; Izquierdo et al., 2014; or Watkins, 2018). An overall analysis should start with an EFA that explores the data and establishes a hypothesis for the factor model in the population. Then, the analysis process should be continued with a CFA to assess if the hypothesis proposed in the exploratory step is plausible in the population. Computing this overall factor analysis seems to be becoming popular among researchers, so here we are interested in how these steps can be planned in terms of the sample to be used in each analysis.

Using the same sample for both EFA and CFA, is obviously an undesirable practice: if the same sample is analyzed using two different methodological approaches, and the outcomes lead to different conclusions, the problem is in the methodological approaches themselves, not in the sample data. If two samples are needed to compute an EFA followed by a CFA, researchers could plan to collect data at two different moments, in two different places, or with two different media. However, all this could introduce biases that lead to non-comparable samples. For example, a sample of individuals collected via Facebook and another collected via TikTok are most likely to be representative of different populations. If different sources to obtain samples can be identified, each sample should not be composed exclusively by participants from a single source. In order to avoid it: (1) all the participants from different sources should be mixed to compose a single large sample; and (2) the large sample should be splat using some method in order to obtain two subsamples. In this way, both subsamples would contain participants from all the sources (see, for example, Del Rey et al., 2021).

In the context of multiple regression analysis, de Rooij and Weeda (2020) point out that there are many ways in which the data can be partitioned in order to compute a cross-validation analysis, and that each set of data can lead to a different regression model. To solve this problem, they recommend repeating the cross-validation several times (with a default of 200 repetitions), and comparing the performance of the different regression models tested among the cross-validation analyses. The proposals by Koul et al. (2018), also focused on multiple regression models, are in the same direction, and they advocate the repetition of the cross-validation study. While this strategy could to some extent be used in factor analysis, so many decisions have to be taken (for example, number of factors to be retained, variables that could be removed from the dataset, linear vs ordinal factor analysis, orthogonal vs oblique models, rotation criterion, essential unidimensional models vs bifactor models, or number of second order factors) that it does not seem to be a very plausible strategy. In the factor analysis context, it would be better to use a split method that aims to produce equivalent dispersion matrices in the different partitioned datasets, so that the cross-validation study is carried out just once.

In the next section, we review the technical options that researchers can use to split a sample in two comparable subsamples in the context of factor analysis. Then, we go on to propose a new method for producing such subsamples. Subsequently, the methods reviewed are compared in two simulation studies: one aims to assess the time taken by different methods to compute equivalent subsamples; and the second aims to assess how the characteristics of the dataset conditions affect method performance. Finally, we apply the methods reviewed and the new one to a different real dataset and assess how they perform.

## Splitting samples in factor analysis

While splitting a sample in half may be seen as unimportant, we shall describe how this apparently innocuous procedure can become more complex than initially expected. We should bear in mind that the key idea is to use an original, single sample to obtain two subsamples that are *equivalent*.

### Strategies available to researchers

Before starting to study the strategies, we must first define what we understand by equivalent samples in the context of factor analysis. Then we will be able to review the strategies that are available.

#### Equivalent subsamples in factor analysis

Factor analysis typically analyzes a correlation matrix: a Pearson correlation matrix in the linear factor model, or a polychoric correlation matrix in the ordinal factor model. In order to assess the suitability of the correlation matrix to be factor analyzed, Kaiser and colleagues proposed the Kaiser-Meyer-Olkin (KMO) statistic (Kaiser, 1970; Kaiser & Rice, 1974). When the index has a zero value, the sum of partial correlations between observed variables is larger than the sum of correlations, which indicates that factor analysis is likely to be inappropriate for use with the corresponding correlation matrix. On the other hand, a value close to one indicates that the sum of partial correlations is not larger than the sum of correlations between observed variables, and that factor analysis should yield distinct and reliable factors. So KMO is related to the common variance in the correlation matrix, and it means that only matrices with high levels of common variance are suitable for factor analysis.

If a sample is divided into two, the subsamples can be regarded as equivalent in the context of factor analysis if all the sources of variance in the original sample are contained in both subsamples. If they are, both subsamples should have a similar amount of common variance, and the KMO value for each subsample will be the same (or very similar). The similarity can be assessed with the following index:

1 $$S=\frac{\min \left({\mathrm{KMO}}_1,{\mathrm{KMO}}_2\ \right)}{\max \left({\mathrm{KMO}}_1,{\mathrm{KMO}}_2\ \right)}.$$

We shall call this index the *Communality ratio* (*S*). If the value of *S* is 1, both KMOs are identical (i.e., the corresponding subsamples are equivalent), while if it is 0, one of the subsamples only contains error variance. So a sample can be considered to be successfully split (i.e., the two subsamples are equivalent), if the *S* value is close to 1.

#### Random splitting of samples

The simplest and most straightforward method for splitting a sample into two halves is to split it at random (see, for example, Mondo et al., 2021). When using this method, our hope is that the random split will send equivalent sets of individuals from the original sample to the subsamples. However, there is no guarantee that this will actually happen. As Osborne and Fitzpatrick (2012) pointed out, large samples made a difference when this procedure was used. In addition, it is also easier to achieve equivalent samples if a large amount of common variance is present (i.e., there are large sets of individuals that share a common profile in the observed variables accounted for by the latent factors in the model). However, researchers frequently have to use relatively small samples in which common variance is not as high as they would like it to be.

If a sample is randomly split into two halves, one of which is analyzed with EFA and the other with CFA, and the conclusions support a well-defined factor model, then the researcher will be glad with the random splitting procedure. However, when things go wrong and the CFA does not confirm the model suggested by the EFA, then the researcher may suspect that the random splitting is to blame for the failure (because it generated non-equivalent samples). The researcher (who would never admit it in the research report) may then be tempted to repeat (again and again) the random splitting of the sample until two halves are obtained that (maybe by chance) match the conclusions of the exploratory and confirmatory factor analyses. Obviously, this would be a perverse use of the split technique, and should never be an option.

For these reasons, it is advisable to use a splitting method that produces equivalent subsamples at once. In this case, if the two equivalent subsamples analyzed using exploratory and confirmatory factor analyses do (or do not) support the same factor model, we will feel more confident that: (a) the result depends on the true factor model in the population (if there is a factor model at all); and (b) the splitting method used to obtain the two subsamples has nothing to do with the conclusions obtained.

#### Methods used in multiple regression

As factor analysis does not seem to have generated a specific method for splitting samples, we need to look for suitable methods developed in other multivariate data analysis techniques. In multiple linear regression, Kennard and Stone (Kennard & Stone, 1969 proposed a method that was later improved by Snee (Snee, 1977), who named it Duplex. The main idea is to generate subsamples of observations that uniformly cover the multidimensional space by maximizing the Euclidean distances between the predictors. Duplex starts by selecting the two elements in the sample that have the greatest Euclidean distance between them and putting them in the first subsample. Then, of the remaining candidates, the two elements farthest from each other are put into the second subsample. In the next step, consecutive elements are selected and put alternatively in the first and second subsamples, the element added being the one farthest away from all the elements already in the subsample. This selection method guarantees the representativeness of the subsamples (i.e., all possible sources of variance are contained in the subsamples).

Duplex can be adapted to the context of factor analysis (see for example, Mas-Herrero et al., 2012; or Morales-Vives et al., 2012). The Euclidean distances between individuals in the sample are computed on the basis of the measured variables. For example, if we are analyzing a psychological test composed of *m* items, the responses of each participant to the *m* items are taken to compute the distances between all the participants. The main drawback is that the datasets in factor analyses need to be so large that Duplex turns out to be almost impracticable. While multiple regression typically requires just a few variables, factor analysis (for example, the items of a psychological test) usually requires a lot, so computing Euclidean distances in the dimensional space defined by the *m* items of a questionnaire is slower in factor analysis than in regression analysis. However, the major difficulty is that distances between all the participants in the sample must be computed and compared a large number of times. For example, in a sample of 5,000 participants, 12,497,500 Euclidean distances need to be computed. Even if each one were computed only once and then stored in the memory of the computer, managing such a large amount of information is not easy. Likewise, adding a new participant to the subsamples requires a compute-intensive task. Our conclusion is that, even if Duplex can be computed in large samples, a faster method should be proposed to optimally split samples in the context of factor analysis.

## SOLOMON: a new proposal for splitting a sample into equivalent subsamples

Our new proposal, which we call Solomon, can simply be regarded as an adaptation of Duplex to the context of factor analysis. In order to explain Solomon, we are going to use an artificial sample of 999 individuals who answered a 5-item questionnaire. We shall focus on the seven participants shown in Table 1.

**Table 1:** Response and distance information for seven participants in the sample

Participants	Participants' responses to items					Projection on components		Distance
	I1	I2	I3	I4	I5	C1	C2	D
Teresa	5	2	1	2	1	2.617	1.140	1.276
Daniel	5	2	1	2	2	2.065	1.094	1.041
Gabriel	3	2	1	5	1	2.540	-1.002	0.869
David	2	2	1	5	1	2.062	-1.324	0.616
Laura	2	5	3	1	4	-0.429	0.759	-0.043
Maria	2	5	4	1	4	-0.937	0.714	-0.260
Carlota	1	2	4	3	3	-1.630	-0.875	-0.823

If we inspect the responses shown in Table 1, we will soon realize that Teresa and Daniel produced a similar response pattern, that Gabriel and David are also similar to each other, and so are Laura and Maria. We will also conclude that Carlota produced a different response pattern to her six colleagues. However, the inspection of the responses will not help us much to describe, for example, how similar Laura’s and Gabriel’s responses are.

The Duplex algorithm computes the 21 distances between these 7 participants in the 5-dimensional space defined by the 5 items. However, in factor analysis we are not interested in the variance contained in the whole *m*-dimensional space, just in the common variance in the lower dimensional space. The first question to be answered is the maximum dimensionality to be considered. In the context of factor analysis, Kaiser proposed the eigenvalue larger-than-one rule to determine the number of dimensions to be interpreted. While this rule is nowadays known to overestimate the number of advisable dimensions, it can be used here just as a conservative bound. In previous studies, I tested the Ledermann bound instead of Kaiser’s rule. This bound is the theoretical maximum number of factors (major plus minor factors) that can be considered in a dataset. However, Solomon performed notably worse when based on the Ledermann bound (especially in datasets with a large number of variables): the presence of variance due to minor factors seemed to introduce more error than useful information in the splitting of the sample. In addition, we would advise to consider at least two dimensions. The most important point is that no *m* dimensions need to be considered, but a much lower dimensionality.

In our opinion, one important feature of a sample splitting method is for it to be independent of the factor model proposed in the subsamples. We propose using Kaiser’s rule because it is well-known to overestimate the number of factors that should be extracted from the dataset at hand. In this regard, Kaiser’s rule seems to be a suitable bound because it makes it possible to include all the sources of variance that account to some extent for the communality in the dataset, but at the same time it avoids having to use as many dimensions as the number of variables (as Duplex does). However, this bound is not meant to be the definitive number of factors to be extracted in the subsamples when a factor model is explored: researchers will have to decide how many factors they actually extract from the correlation matrix in order to propose a factor model.

**Fig. 1:**
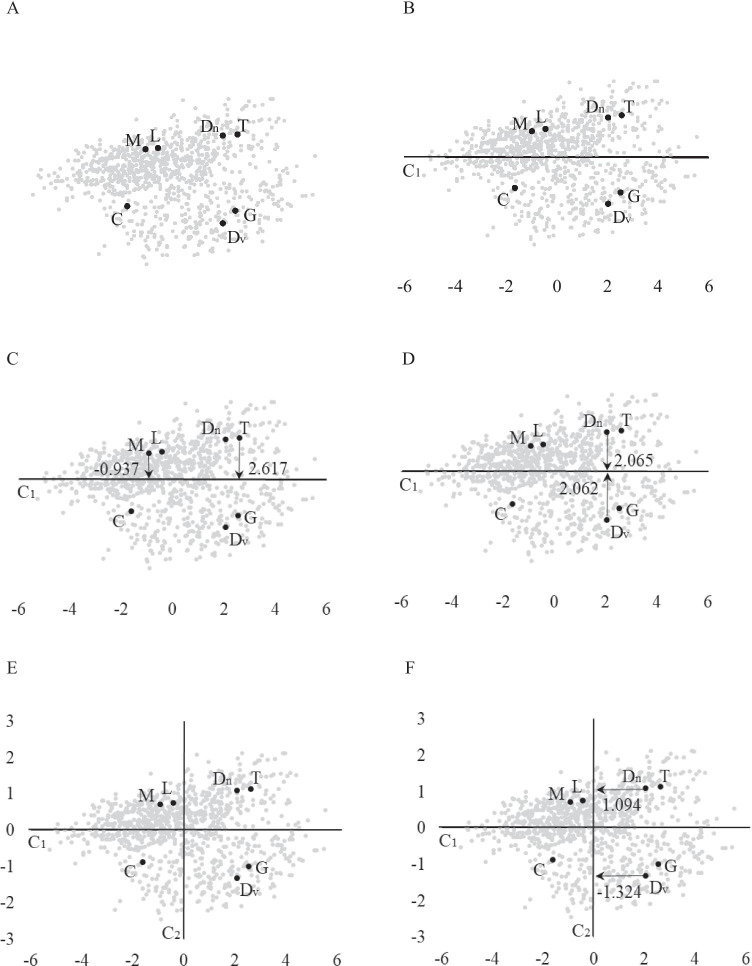
Graphical representation of individuals in a bidimensional space

In the case of our example with *m*=5, the maximum number of factors related to the common variance that we shall consider is 2. This bidimensional space can be graphically represented in a plot, in which individuals are represented as a cloud of points. Panel A in Fig. 1 represents this plot. Each of the 999 participants are represented by a point, and the seven participants in Table 1 have been highlighted with a bold point so that they can be clearly identified in the cloud. The visual inspection of this cloud of points is even more informative than the five response scores in Table 1. It is now easy to see how different Laura (L) and Gabriel (G) are.

So far we have simplified the dimensionality of the space. Instead of considering an *m*-dimensional space, we shall just consider a *Q*-dimensional space, where *Q* is always lower than *m*.

Duplex now computes Euclidean distances between the 999 individuals in the sample: this involves 498,501 distances. Instead of this, we propose computing a single numerical value that represents the position of each of the 999 participants in the cloud. To do this, we can set a reference and describe participants’ positions with respect to this reference. We propose that this reference be the principal components. Principal Component Analysis (PCA) is closely related to factor analysis: PCA aims to summarize the variance in the variables involved, while factor analysis aims to identify the dimensional model related to the common variance.

If we compute the component scores of each participant, the first component in the canonical PCA solution is an axis that crosses the cloud of participants through the center: Panel B in Fig. 1 represents the first component. Now the projection of each participant on the first component helps us to describe its position in the cloud of participants. Column C_1_ in Table 1 displays these projections, and Panel C shows Teresa’s and Maria’s projections on the axes: we can see that they are some distance apart in the cloud, and their projections (2.617 and -0.937, respectively) help to describe the difference between them. However, considering only the projection on the first component can be misleading. In Panel D, we can see that Daniel (Dn) and David (Dv) are not close together in the cloud of participants. However, their projections on the axis (2.065 and 2.062, respectively) would suggest that they are very similar. The reason for this is that we take into account only the first component, while Kaiser’s rule advises for this dataset to consider two dimensions.

The second component analysis (also in the canonical position) is again an axis that crosses the cloud of participants through the middle, but which is orthogonal to the first component. Panel E presents the axes related to the two components. We can now clarify that the graphical plot was from the very beginning represented in these two axes. The participants’ projections on the second component are presented in column C_2_, and Panel F shows Daniels’ and David’s projections, which reveal (as we have already seen in the plot) that they are not so close in the cloud of participants.

In conclusion, we need to consider the projection on both axes in order to effectively describe the participant’s position. However, our aim was to have a single numerical value to describe each participant’s position, and we ended with two values (i.e., the projection on each component). One solution to this is to combine the two numerical values into one. However, directly adding one to the other would not be the best idea because this means that we consider the projections on both components to be equally important in describing the participant. Each component is related to a different amount of variance. In our example the first component accounted for 41.1% of the variance while the second accounted for 17.5%, which means that we should give much more importance to the projection on the first component than the projection on the second. To obtain a single numerical value that describes participants’ position in the cloud we can compute a weighted addition:

2 $$d_i={\textstyle\sum_{j=1}^Q}w_jc_{ij},$$

where *w*_*j*_ is the proportion of variance accounted for by the *j-*th component, *c*_*ij*_ is the projection of the *i*-th participant on *j*-th component, and *d*_*i*_ is the numerical value that describes the position of the *i*-th participant in the cloud of participants. Column D in Table 1 orders the seven participants highlighted in the example in terms of this numerical description. The further two participants are from each other in the sorted list, the more different they are. Teresa and Carlota are the two individuals who are furthest apart in the sorted list, and we can see in Fig. 1 that they are at some distance from each other. It should be said that this distance has to be computed for the 999 participants in the sample.

Now that the participants in the sample are ordered in terms of their position in the cloud of participants, the two subsamples can easily be computed: odd-numbered participants are assigned to subsample one, and even-numbered participants to subsample two. As the assignment to each subsample is made for the whole sample, from the information in Table 1 it is impossible to know to which subsample the seven highlighted participants will be assigned. However, just for the benefit of the pedagogical explanation, let’s suppose that 992 participants have already been assigned to their subsamples, and the seven participants in our example are the last ones to be assigned. Teresa, Gabriel, and Laura would be assigned to the first subsample, and Daniel, David, and Maria to the second. However, we have an uneven number of participants so what should we do with Carlota? In fact, she could be assigned to either subsample: a single participant should not make a difference to the subsamples if the number of participants is large enough. For the sake of consistency, we should assign Carlota (an odd-numbered participant), to the first subsample.

Our aim was to obtain a splitting method that was faster than Duplex, but what we found also needs a considerable amount of computing. However, our Matlab code for Solomon did manage to split a large sample substantially faster than Duplex and it can easily be computed using any statistical software that includes PCA. All that is needed is to obtain participants’ component scores in a *Q*-dimensional solution, and to compute the weighted sum (*d*_*i*_) using the proportion of variance related to each component. Then, participants must be sorted in order of their *d*_*i*_ value. Finally, odd-numbered participants are assigned to the first subsample, and even-numbered participants to the second subsample.

Of course, it still remains to be seen which of the two methods provided the most equivalent subsamples in terms of Communality Ratio (index *S*). In addition, as Solomon’s most important characteristic is the short computing time needed to obtain equivalent subsamples, the time taken by Duplex and Solomon to split large samples should be compared.

## First simulation study

The aim of the simulation study is to compare the time taken by Duplex and Solomon to compute equivalent subsamples. We were not interested in assessing the real computation time, because it can vary considerably from one computer to another. Instead, we were interested in the comparison when the two methods were computed with the same computer. In addition, for the time estimates to be realistic, the simulation was carried out on a laptop. Laptops are popular among applied researchers, but are not very efficient at large computing tasks.

### Study design

The simulation study was computed with Matlab. The Duplex method was computed with the code proposed by Daszykowski et al. (2002), which is the fastest implementation that we know in Matlab. Solomon was computed with the code we produced ourselves.

The most challenging aspect of splitting a sample into equivalent subsamples is the size of the sample itself. For this reason, this was the characteristic that we manipulated in the simulation study. In order to generate sample data, we produced a population loading matrix of 5 factors, and 20 variables for each factor. Salient loading values of the variables were uniformly chosen in the range [.40, .45], while non-salient loading values were uniformly chosen in the range [-.10, .10]. From the population loading matrix, the corresponding population correlation matrix was obtained. Then, a normal random sample was obtained that had the population correlation matrix obtained in the previous step. The random samples were generated with sample sizes in the range [500, 7,500] with steps of 500 (i.e., samples of *N* equal to 500, 1,000, 1,500, …, 7,500]. Each sample was split using the Duplex method and the Solomon method.

We recorded the time taken by each method to split the sample. In addition, to assess the quality of the equivalence between subsamples, we computed the *S* index. Our expectation was that both methods would provide equivalent subsamples with similar *S* indexes.

We replicated the simulation process 100 times, so a total of 1,500 samples were split in half during the simulation study.

### Results of the first simulation study

We computed the mean and standard deviation of the *S* index and the time taken to split the different sample sizes (see Table 2 and 3).

**Table 2:** Mean and standard deviation for the comparison between Duplex and Solomon in the simulation study

Sample size	DUPLEX		SOLOMON	
	Computing time	*S*	Computing time	*S*
500	0.7274 (0.0568)	.9797 (.0160)	0.0075 (0.0019)	.9731 (.0189)
1,000	1.9661 (0.0817)	.9912 (.0067)	0.0082 (0.0015)	.9881 (.0085)
1,500	6.0192 (0.0957)	.9941 (.0046)	0.0106 (0.0033)	.9919 (.0053)
2,000	12.5072 (0.1767)	.9957 (.0035)	0.0113 (0.0020)	.9953 (.0039)
2,500	22.1769 (0.5978)	.9961 (.0034)	0.0122 (0.0022)	.9960 (.0033)
3,000	34.8135 (0.3744)	.9970 (.0024)	0.0133 (0.0026)	.9955 (.0031)
3,500	51.6800 (0.3214)	.9976 (.0018)	0.0142 (0.0024)	.9967 (.0025)
4,000	73.7489 (0.4880)	.9980 (.0017)	0.0154 (0.0026)	.9973 (.0021)
4,500	100.7430 (0.6465)	.9981 (.0012)	0.0171 (0.0026)	.9975 (.0020)
5,000	133.6307 (0.7956)	.9984 (.0012)	0.0185 (0.0027)	.9979 (.0017)
5,500	172.5596 (0.9153)	.9984 (.0013)	0.0201 (0.0046)	.9981 (.0013)
6,000	219.2720 (0.9102)	.9984 (.0012)	0.0207 (0.0073)	.9983 (.0014)
6,500	274.7532 (1.0997)	.9984 (.0011)	0.0207 (0.0070)	.9983 (.0012)
7,000	336.2905 (1.6212)	.9986 (.0010)	0.0207 (0.0064)	.9985 (.0012)
7,500	414.2110 (5.8583)	.9989 (.0008)	0.0232 (0.0065)	.9985 (.0011)

computing time is expressed in seconds

**Table 3:** Mean of *S* indices obtained in the second simulation study using three sample splitting methods. Standard deviations are given in parenthesis. (The largest mean per condition is printed in bold)

Condition	Random Sampling	Duplex Splitting	Solomon Splitting
Overall	.9578 (.0657)	.9600 (.0749)	**.9658** (.0576)
*h* = large	.9650 (.0608)	.9629 (.0817)	**.9722** (.0520)
*h* = wide	.9505 (.0695)	.9571 (.0674)	**.9594** (.0619)
*r* = 1	.9574 (.0621)	.9549 (.0901)	**.9714** (.0461)
*r* = 2	.9579 (.0674)	**.9640** (.0625)	.9635 (.0609)
*r* = 3	.9581 (.0678)	.9615 (.0671)	**.9618** (.0649)
*m/r* = 10	.9551 (.0585)	.9610 (.0612)	**.9631** (.0511)
*m/r* = 20	.9591 (.0677)	.9587 (.0818)	**.9669** (.0590)
*m/r* = 30	.9594 (.0710)	.9603 (.0812)	**.9676** (.0627)
*N* = 100	.8779 (.1002)	.8849 (.1186)	**.8987** (.0881)
*N* = 200	.9303 (.0719)	.9355 (.0841)	**.9420** (.0672)
*N* = 400	.9735 (.0259)	.9759 (.0425)	**.9801** (.0200)
*N* = 800	.9870 (.0145)	.9867 (.0318)	**.9904** (.0109)
*N* = 1,600	.9932 (.0083)	.9918 (.0250)	**.9951** (.0060)
Normal responding	.9677 (.0559)	.9729 (.0521)	**.9734** (.0501)
Extreme responding	.9478 (.0729)	.9471 (.0905)	**.9582** (.0633)

Values printed in bold are the largest mean value of index S for each condition

*h*: communality level; *r*: number of factors; *m*: number of variables.

As can be observed, the equivalence of the subsamples in terms of the *S* index was slightly worse when small samples were split (between 500 and 1,500), and values were best with Duplex. However, when samples were large, these differences disappeared.

**Fig. 2:**
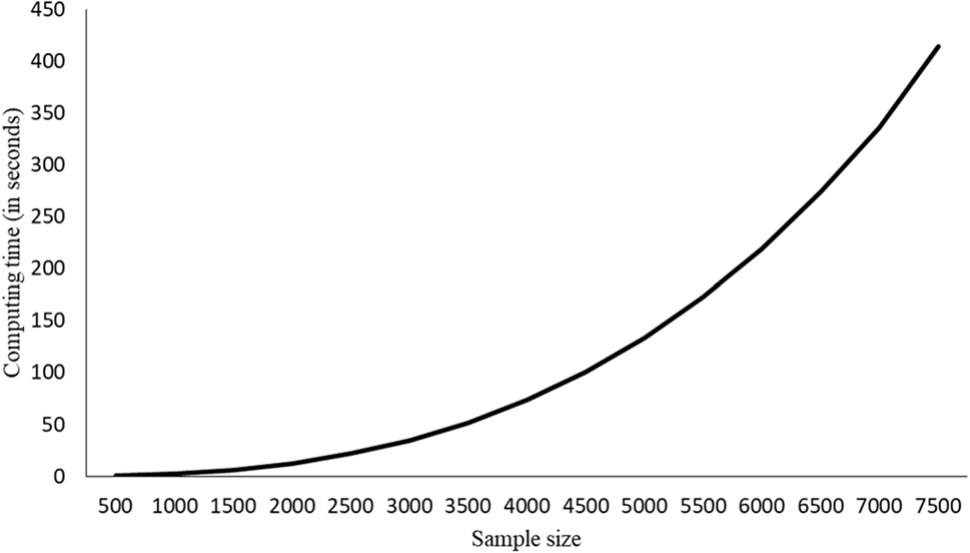
Computing time needed by Duplex in the simulation study

The computing times needed by Duplex ranged between 0.63 seconds (samples with *N*=500) and 7.30 minutes (samples with *N*=7,500). Figure 2 shows how the time increased as the samples got successively larger. With samples of *N*=4,000, already more than a minute was required.

On the other hand, the computing times taken by Solomon ranged between 0.003 seconds (samples with *N*=500) and 0.059 seconds (samples with *N*=7,500). Solomon’s computing time for the largest samples was still less than the time needed by Duplex for the smallest.

Finally, although the *S* index was systematically larger with Duplex, the outcomes in Table 2 show that the differences are so small that the outcomes with Solomon can be regarded as equivalent when samples were larger than 1,000.

## Second simulation study

The simulation study intended to assess the extent to which the random splitting of samples, the Duplex method and the Solomon method provide equivalent subsamples in terms of Communality Ratio (index S). The assessment was designed for various scenarios with different communality levels, number of variables per factor, number of factors in the population, sample size, and response extremeness. In general terms, the design attempted to mimic the conditions expected in empirical applications, and so provide realistic choices.

### Study design

We specified two levels of communality in the population model (large: salient loadings randomly and uniformly drawn from the range [.55 - .88]; and wide: salient loadings randomly and uniformly drawn from the range [.20 - .88]). There were 1, 2 and 3 factors in the population and 10, 20, and 30 variables per factor. For each factor model at hand, a population of 10,000 continuous responses were simulated following a normal distribution, and the simulated responses were categorized to a 5-point response format so that there were two response style conditions: normal distributed responding (the thresholds used to categorize data were [.05, .26, .74, .95]; and extreme responding (the thresholds used to categorize data were [.05, .10, .15, .25]). Extreme responding represents situations in which responses are mainly in one of the extreme response categories, and few participants use the whole range of categories: this is quite usual for psychological tests.

From each population of responses, samples of different sizes were uniformly drawn. The sizes of the samples were: 100, 200, 400, 800, and 1,600. Once these samples became available, they were split into two subsamples using the three methods assessed, and the S index was computed for each pair of subsamples.

To summarize, the study was based on a 2×3×3×5×2 design with 1,000 replicas per condition. The independent variables were: (1) communality: wide and high; (2) number of factors in the population model: from 1 to 3; (3) number of variables per factor (10, 20, and 30); (4) size of samples (100, 200, 400, 800, and 1,600); (5) response style: normal distributed responding, and extreme responding. To avoid unrealistic situations, the study was not fully crossed: for example, with the conditions *N*=100, *r*=3, and *m/r*=30 (where,*r* is the number of factors, and *m* is the number of variables), the number of measured variables (i.e., responses to items) would be 90, while for the subsamples (i.e., the number of participants) it would be 50. This is an unrealistic situation in applied research. In total, the number of samples generated and split during the study was 168,000.

### Results of the second simulation study

The first outcome of note was that the Duplex splitting algorithm failed to converge on 137 occasions (0.08%), and no optimal splitting was reported. These failures were quite systematic when data sets were being analyzed in which the population factor model was unidimensional, the communality high, and the response style extreme responding. With these 137 samples, random sampling and Solomon had no difficulties, and obtained subsamples in which the mean of the *S* index was .875 and .922, respectively. These problematical samples are not included in the analysis reported below.

In general, random sampling was the method that performed worse. It should be said that its performance was hardly affected by the independent variables, except sample size and response style. When sample size was large (N equal to 800 or larger), it performed slightly better than Duplex. In samples in which the response style was extreme responding, it clearly performed less well (but still slightly better than Duplex splitting). It performed worse in small samples.

In general, Duplex was between the other two methods, but variability was larger, which makes the method a bit unreliable: that is to say, it provided optimally equivalent subsamples, and, at the same time, the least equivalent subsamples. The most favorable situations for Duplex were when it had to deal with samples that were large, and when the response style was normal responding. When the response style was normal distributed responding, it also performed well.

Solomon splitting performed systematically better than the other two methods. In comparison, it seems the best option when samples are related to a unidimensional factor model, when communalities are high, when samples are small, and when the response style is extreme responding. It is also always the method that shows the lowest levels of variability, which means that it is the most reliable option.

## Analyses of real datasets

In this section, we study the performance of the random splitting method, Duplex and Solomon in twelve real datasets, all of which have a sufficiently large sample.

### Datasets

The twelve datasets analyzed by our research group over the last thirty years are revisited for the purposes of this study. Most of the samples are the same as the ones used for previous publications. In some cases, we have done further work with the related test, and the sample used here is larger than when first published. We aimed to collect datasets with different numbers of items (between 9 and 100). In addition, we wanted the number of factors selected for the factor model to be in a wide range (1 to 7 factors). The characteristics of the datasets are the following:

1. MSPSS is a 12-item instrument that measures the perceived adequacy of social support (for details see Calderón et al., 2021). The study sample comprised 925 patients with cancer (60.3% females), aged between 24 and 85 years (Mean: 59.0; Standard deviation: 12.2).
2. BAI is 24-item test that was developed to assess belief in astrology (for details see Chico & Lorenzo-Seva, 2006). The participants were 743 undergraduates studying Psychology and Social Sciences at university (84.1% females), aged between 18 and 60 years (Mean: 21.7; Standard deviation: 4.3).
3. RAS is a Spanish version of the Reducer-Augmenter Scale that has 61 items (for details see Piera et al., 1993). There were 1,156 participants (37.2% females), aged between 16 and 53 years (Mean: 21.2; Standard deviation: 4.2).
4. SDMQ is a 9-item instrument that assesses the perspective of physicians and how they share decision making with patients (see Calderón et al., 2021). It has two dimensions: (1) the information and explanations given by the physician, and (2) the choice of the best treatment option for the patient. The sample consisted of 520 individuals (67.1% female), aged between 26 and 85 years (Mean: 59.2; Standard deviation: 12.2).
5. SAS is a 24-item instrument that assesses statistical anxiety (for details see Vigil-Colet et al., 2008). The test has three scales: (1) examination anxiety, (2) asking for help anxiety, and (3) interpretation anxiety. There were 459 participants (76% females), aged between 18 and 55 years (Mean: 21.6; Standard deviation: 3.5).
6. EPIA is a 57-item inventory from the Spanish validation of the Eysenck Personality Inventory, which measures personality. It has three scales: (1) extraversion-introversion, (2) neuroticism-stability, and (3) social desirability. The sample was collected during the study by Piera et al. (1993) for purposes of validity, and consisted of 756 participants (24.2% females), aged between 16 and 53 years (Mean: 20.7; Standard deviation: 3.6).
7. BSWQ is a 12-item questionnaire for the self-assessment of individual differences in language switching (for details see Rodriguez-Fornells et al., 2012). The test has four scales: (1) L1-Switch, which measures the tendency to switch to Spanish (L1); (2) L2-Switch, which measures the tendency to switch to L2 (Catalan); (3) contextual switch, which indexes the frequency of switches in a particular situation or environment; and (4) US, which measures the lack of awareness of language switches. The participants were 582 Spanish–Catalan bilingual university students (75.1% women) with a mean age of 21.7 (3.5) years.
8. PSYMAS is a 25-item questionnaire that assesses psychological maturity in adolescents and consists of three subscales: (1) work orientation, (2) identity, and (3) autonomy (for details see Morales-Vives et al., 2012). The participants in the study were 691 high school students (56.5% females), between 15 and 18 years old (Mean: 16.5; Standard deviation: 0.9).
9. I-DAQ is a 27-item questionnaire that measures aggressive behaviors and has five factors: (1) physical aggression, (2) verbal aggression, (3) indirect aggression, (4) social desirability, and (5) acquiescent responding (for details see Ruiz-Pamies et al., 2014). There were 882 participants in the present study (61.7% females), between 18 and 68 years old (Mean: 27; Standard deviation: 7.2).
10. MBRQ is a 22-item questionnaire that measures the musical reward experience and can be decomposed into five reliable factors: (1) musical seeking, (2) emotion evocation, (3) mood regulation, (4) social reward, and (5) sensory-motor (for details, see Mas-Herrero et al., 2012). The questionnaire was administered via an internet application to 758 participants (53 % females, and 14 % professional musicians) who responded voluntarily (age range: 18-78 years old (Mean: 33.9; Standard deviation: 10).
11. FFPI is a 100-item test that measures personality traits. It has six dimensions: (1) extraversion, (2) agreeableness, (3) conscientiousness, (4) emotional stability, (5) openness to experience, and (6) acquiescent responding (for details see Rodríguez-Fornells et al., 2001). The Spanish sample consisted of 567 undergraduate college students (84.7 females) enrolled in an introductory psychology course. The mean age for this group was 19.3 years (Standard deviation: 2.8).
12. OPERAS is a 40-item personality test that has seven dimensions: (1) extraversion, (2) agreeableness, (3) conscientiousness, (4) emotional stability, (5) openness to experience, (6) acquiescent responding, and (7) social desirability (for details see Vigil-Colet et al., 2013). The participants in the present study are 5,503 (52.1% females), and they were between 11 and 95 years old (Mean: 31.3; Standard deviation: 14.8).

### Methods compared

For each of the twelve samples, we computed how the three methods split the samples. In order to assess how equivalent the subsamples were, we computed the Communality Ratio (i.e., *S* index) described above. We carried out the random split method 10 times for each sample and then the mean and standard deviation of the values of index *S*.

### Results

Table 4 shows the main characteristics of the samples discussed, plus the KMO of the overall sample. For the random sampling method, the table shows the mean and the standard deviation of the values of index S after the 10 trials. For Duplex and Solomon, the table shows the KMO indices obtained in each subsample, and the *S* index.

**Table 4:** KMO indices obtained in the illustrative datasets after using three sample splitting methods. (The maximum value of index S for each sample is printed in bold.)

Scale	*r*	*m*	*N*	Total sample	Random splitting (*S*)		Duplex Splitting			Solomon Splitting		
					Mean	*Sd*	First half	Second half	*S*	First half	Second half	*S*
MSPSS	1	12	925	.8685	.9683	.0207	.8531	.8591	.9930	.8594	.8615	**.9975**
BAI	1	24	743	.9435	.9891	.0091	.9333	.9372	**.9958**	.9304	.9360	.9940
RAS	1	61	1,156	.8354	.9825	.0090	.8187	.8271	.9898	. 8203	.8227	**.9971**
SDM	2	9	520	.8722	.9508	.0280	.8538	.8813	.9688	.8606	.8705	**.9886**
SAS	3	24	459	.9298	.9827	.0082	.9084	.9242	.9829	.9118	.9153	**.9961**
EPIA	3	57	756	.7815	.9643	.0123	.7515	.7658	**.9813**	.7403	.7654	.9672
BSWQ	4	12	582	.8381	.9700	.0191	.8100	.8126	**.9968**	.8006	.8152	.9821
PSYMAS	5	25	691	.7489	.9601	.0283	.7117	.7396	.9623	.7087	.7363	**.9625**
I-DAQ	5	27	882	.8576	.9733	.0118	.8311	.8425	.9865	.8355	.8356	**.9999**
MBQR	5	22	758	.8456	.9665	.0197	.8336	.8373	.9956	.8273	.8288	**.9983**
FFPI	6	100	567	.8880	.9770	.0112	.8069	.8172	**.9874**	.7937	.8233	.9641
OPERAS	7	40	5,503	.8894	.9939	.0045	.8828	.8881	.9941	.8833	.8842	**.9991**

Values printed in bold are the largest value of index S for each sample analysed

*r*: number of factors; *m*: number of items; *N*: Sample size; *S*: Communality ratio index

In most of the samples (11 out of 12), the KMO indices of the subsamples were lower than the KMO value of the original sample. This means that, as individuals with a response pattern that best accounted for the common variance are accurately distributed among the subsamples, these individuals are not so well represented in the subsamples (at least not as they were in the original sample), and the KMO value of each subsample is lower than in the original sample. The only sample in which the subsamples had a KMO value similar to that of the original sample (no difference until the third decimal digit) is the one for OPERAS, which was so big (*N*=5,503 individuals) that even after distributing the best individuals between the samples, there were still so many of them in each subsample that the KMO value remained unchanged. The conclusion is that very large samples help to obtain equivalent subsamples with KMO values similar to the KMO value of the whole sample.

The outcomes of the random splitting method show that although optimally equivalent subsamples in terms of S were obtained in each dataset, non-optimally equivalent subsamples were also obtained. When subsamples are not optimally equivalent, one of the subsamples can have a KMO index that is even larger than that of the total sample, while the other subsample has a KMO index that is very low. For example, the KMO value of the SDM dataset was .8722, while some of the KMO values observed for subsamples were .7481 and .9004, respectively. This means that one of the samples was assigned most of the individuals with a response pattern that best accounted for the common variance, while the other subsample was largely assigned the individuals with response patterns due to specific variance. The *S* index values were worse than those obtained by the other methods.

The Solomon method provided more equivalent subsamples than Duplex: in 8 samples (out of 12), the *S* index showed the best outcome. A *t*-Student test for dependent samples was conducted in order to test the difference between (1) the mean of S values obtained in the 10 times random splitting, and (2) the S values obtained by Solomon. Differences were significant in comparison to those obtained with random splitting (*t*-Student=4.40; *P* < .01).

Finally, Solomon performed slightly better than Duplex in terms of the *S* index (means of .987 and .986, respectively). In addition, the Solomon method still provided the best *S* values on eight occasions. It should be pointed out that Solomon performed best with the two largest samples. As the differences between Duplex and Solomon were not significant (*t-*Student=0.499; *P* = .628), all the conclusions drawn for Duplex can also be drawn for Solomon.

## Solomon implementation in statistical packages

We implemented Solomon method in three different statistical programs, and made it available at the web page of our university (http://www.psicologia.urv.cat/en/tools/). The utilities developed are:

1. The R script “solomon.r”. It is a script that uses only native functions in R, so no packages needs to be downloaded to use it. In order to use it, the researcher has to store participants’ responses in a text file, update the name of the input and output files, and to execute the script. In the output file, the first column indicates the assignment of each row to one or the other subsample.
2. The SPSS script “solomon.sps”. Again, in order to use it the researcher must have participants’ responses in a SPSS data file, and to execute the script. A new data file is generated with the first variable indicating the assignment of each row.
3. Finally, we implemented Somolon method in our program to compute factor analysis, that can be downloaded free from the site (http://www.psicologia.urv.cat/media/upload/domain_2082/arxius/Utilitats/factor/index.html). To help the researcher to use Solomon method insight FACTOR, a video tutorial is also available at the web site.

## Discussion

We studied three methods for splitting samples into two halves. The most popular one nowadays (the random splitting method) is based on the hope that a random procedure will help to provide two equivalent samples.

The first simulation study showed that Solomon is the quickest at splitting samples, and that it takes substantially less time than Duplex when the sample is large. The second simulation study revealed that Solomon generally provided the best optimally equivalent samples, and the lowest variability. It must be said that all the methods gave acceptable results when the sample was large. At the same time, the most difficult situation to deal with is when the response style is extreme responding. When it is, most of the participants in the sample show similar responses around a few response categories, and at the same time some participants use categories at the other end of the response scale. With such complex samples, Solomon was the most accurate method.

In our study with real datasets we showed that even when equivalent samples were obtained with Duplex and Solomon, random splitting can provide subsamples that are not so optimally equivalent in terms of the quality of the correlation matrices. Of course, random splitting could be used differently from the way we used it: a number of subsamples could be randomly split until a high value of Communality ratio index (*S*) value is obtained. However, this approach is not optimal from the point of view of computing time. In addition, even when a reasonable *S* value is obtained in this way, there is no way of knowing if a better split could be obtained with the data.

If the advice given by de Rooij and Weeda (2020) in the context of multiple regression analysis is adapted to factor analysis, researchers may: (1) repeat the random splitting of the sample several times (they use 200 repetitions as the default); (2) assess the cross-validation of the factor model in each repetition; and (3) compare the performance of the different factor models tested in different cross-validation analyses. While not impossible, however, this approach does not seem very practical in the context of factor analysis.

Duplex is a method that was developed in the context of multiple regression analysis. While it can be used in the context of factor analysis, the outcome of our simulation study shows that it is very slow when the sample is large. And the analysis of real data shows that samples need to be large to obtain subsamples that are not just equivalent with each other, but which also have a KMO index similar to the one in the original sample. For these reasons, the method does not seem to be truly optimal in the context of factor analysis.

Solomon, which is well adapted to the context of factor analysis, is our alternative proposal for splitting samples in such a way that subsamples are equivalent. It is also fast. The simulation study shows that it is much faster than Duplex, and that the sample size does not substantially impact its performance. In addition, the second simulation study and the analysis of real datasets show that Solomon provides subsamples that are as equivalent as the ones provided by Duplex. It must be said that, when analyzing the set of real datasets, the equivalence of the samples provided by Solomon was slightly better, but the difference was not significant.

Our explanation describes how Solomon can be used to provide two equivalent subsamples. However, in some situations researchers aim to have more groups. For example, Davies et al. (2021) divided their sample of *N*=2,033 participants into three. Solomon can be adapted to obtain more than two subsamples. When there is an even number of subsamples, Solomon has to be applied a number of times: first, it has to be applied to the main sample; and, second, it has to be applied to each subsample as many times as needed in order to obtain the number of subsamples required. If there is an odd number of subsamples, then our procedure can be adapted slightly. For example, if three subsamples are required (as was the case in Davies et al., 2021), once the individuals are sorted by their value in *d*_*i*_, triplets of consecutive individuals should be selected and each participant in the triplet assigned to a different sample.

In other situations, external variables are taken into consideration. For example, Del Rey et al., (2021) randomly split their sample into two halves, controlling for the gender variable. This can also be done using Solomon: the sample should the split into two groups by gender, and these two samples then split again using Solomon to obtain two equivalent subsamples of women, and two equivalent subsamples of men. Subsequently one subsample of women and one subsample of men should be joined to form a single subsample, and the process repeated with the other two subsamples to form a second single subsample. For complex controlling variables that involve more than two groups of individuals (for example, if an individual has to be assigned to a subsample of one of the groups defined by the variable LGBTI), the procedure would be similar. In this case, however, it would be advisable to have a large sample in which the proportion of individuals in each group of the controlling variable is representative of their proportion in the population.

Our aim was to propose a splitting method that is independent of the factor model proposed by the researcher in the subsamples. As a bound of the dimensions considered to split the dataset, we have proposed Solomon based on Kaiser’s rule: this means that the sample is split on the basis of the variance due to the number of dimensions that Kaiser’s rule suggests. As this is an arbitrary decision, the researcher could propose other bounds. For example, if the researcher has an idea of how many factors will be extracted for the factor model (based on previous research, or empirical evidence), then this number of factors could be used to split the dataset. Whatever the number of dimensions used by the researcher, however, we would advise not to use more factors than the ones suggested by Kaiser’s rule.

A final word about sample size. As Osborne and Fitzpatrick (2012) pointed out, sample size is important in studies that focus on subsamples. Our analysis of real datasets showed that the value of the KMO in the original sample is only maintained if the equivalent subsamples are also large. If a large number of subsamples is required, then the size of the whole sample must also be very large. In addition, if the analyses of the different subsamples converge to the same conclusions (i.e., the acceptance of a particular factor model in the population), it would be advisable to join the subsamples again in order to estimate the parameters of the model. When estimating factor reliabilities and scores, for example, it is advisable to use a sample that is as large as possible. The reason for this is that these estimates also need a considerable number of factor model parameters in the population to be estimated (this is the case of the ORION reliabilities and scores, Ferrando & Lorenzo-Seva, 2016). All these estimates will be more stable, and therefore more credible, if they are based on the largest sample the researcher has available (that is to say, the whole sample).

Solomon should be easy to implement in statistical packages as long as principal component analysis is available. The example that we provide in our page site in R and SPSS should help other researchers to implement it in other packages.
